# COVID-19 symptoms, internet information seeking, and stigma influence post-lockdown health anxiety

**DOI:** 10.3389/fpsyg.2023.1228294

**Published:** 2023-08-10

**Authors:** Qian Li, Xue Yang, Xin Wang, Han Zhang, Ningning Ding, Wenqian Zhao, Wenwen Tian, Jiankang He, Mingxuan Du, Haiyan Hu, Guohua Zhang

**Affiliations:** ^1^Faculty of Medicine, Centre for Health Behaviours Research, Jockey Club School of Public Health and Primary Care, The Chinese University of Hong Kong, Shatin, Hong Kong SAR, China; ^2^School of Mental Health, Wenzhou Medical University, Wenzhou, China; ^3^Zhejiang Provincial Clinical Research Center for Mental Disorders, The Affiliated Wenzhou Kangning Hospital, Wenzhou Medical University, Wenzhou, China

**Keywords:** health anxiety, internet information seeking behavior, stigma, COVID-19, mediation

## Abstract

**Background:**

With the lifting of Zero-COVID policies in China, rapid transmission of the virus has led to new challenges for patients’ health anxiety. This study aimed to evaluate the relationship between COVID-19 symptoms and health anxiety, as well as the mediation paths between them in individuals infected with COVID-19.

**Method:**

A cross-sectional study was conducted in December 2022, following the relaxation of anti-COVID measures in China. A validated online questionnaire was used to collect data from COVID-19 patients on the number and severity of symptoms, health anxiety, internet health information-seeking behavior (IHISB), and perceived stigma. Structural equation modeling was used to examine the mediation model in which COVID-19 symptoms would affect health anxiety via IHISB and perceived stigma.

**Results:**

Overall, 1,132 participants (women, 67.6%) were included, with a mean (SD) age of 28.12 (10.07) years. Participants had an average of seven COVID-19 symptoms, with cough (91.3%), nasal congestion (89.1%) and fatigue (87.8%) being the most common. The number and severity of COVID-19 symptoms, IHISB, perceived stigma, and health anxiety were positively correlated with each other after adjusting for covariates (r ranging from 0.10 to 0.81, all *p* < 0.05). IHISB (effect = 0.14, *p* < 0.001) and perceived stigma (effect = 0.04, *p* < 0.001) fully mediated the relationship between COVID-19 symptoms and health anxiety.

**Conclusion:**

Interventions for health anxiety reduction during and after pandemics should target improving the quality of online health information, enhancing individuals’ online healthy literacy, and reducing stigma.

## 1. Introduction

The Coronavirus disease 2019 (COVID-19) pandemic has had profound negative impacts on human health, well-being, as well as economic and social systems (Vancini et al., 2022; Taheri et al., 2023). According to World Health Organization (WHO), there have been over 761 million confirmed cases of COVID-19 and over 6.8 million recorded deaths globally in 2023 (WHO, 2023a). As the COVID-19 pandemic enters its fourth year, some countries have started to ease stringent restrictions that were implemented during the initial phase (Han et al., 2020). On December 7th, 2022, the State Council of China issued new guidelines to ease Zero-COVID policies (Mallapaty, 2022). A rapid increase in infection rates of COVID-19 from 5.7 to 80.7% occurred in a short period, which placed a strain on hospital resources, including staff and beds (Huang et al., 2023). Most people were self-diagnosed and self-care at home.

Health anxiety is characterized by distressing emotions, physiological arousal, and bodily sensations that stem from perceived threats to one’s general health (Asmundson et al., 2010). This phenomenon is frequently observed among individuals who have contracted COVID-19, particularly during the phase of easing anti-COVID measures (Akbari et al., 2021). Health anxiety has been well documented to have detrimental effects on various aspects of an individual’s life, such as work performance, family involvement, help-seeking, recovery, and physical health [e.g., (Asmundson et al., 2010; Jungmann and Witthöft, 2020; Haig-Ferguson et al., 2021)]. It is prevalent in patients with emerging or severe infectious diseases, such as severe acute respiratory syndrome (SARS), Middle East respiratory syndrome coronavirus outbreak (MERS), and acquired immunodeficiency syndrome (AIDS) (Rachman, 2012; Baker et al., 2022). We did not identify any studies on whether the number and severity of COVID-19 symptoms would affect anxiety about general health. To prevent health anxiety and related adverse social and health outcomes of people who have contracted COVID-19, it is essential to identify potential mediating factors that can break the link between COVID-19 symptoms and health anxiety.

The internet and social media platforms became the key sources of health-related information for infected people in the recent wave of COVID-19 (Neely et al., 2021). The *Protection Motivation Theory* (Rogers, 1975) argues that when people perceive a threat as high and feel that they are unable to cope with it, they are more likely to engage in protective behaviors. Thus, being infected would trigger individuals’ threat-appraisal and coping-appraisal processes. Perceiving greater or more symptoms of COVID-19 may lead to more motivation and behaviors of seeking information to reduce their uncertainty about their physical condition and the disease. Empirical studies have demonstrated that patients with diabetes and cancer are likely to search for related health information through the Internet (Xiao et al., 2014; Jamal et al., 2015). However, seeking information online may place more attention on the feared illness and serve to maintain health anxiety (Salkovskis and Warwick, 1986; Salkovskis and Warwick, 2001; McMullan et al., 2019). According to the cognitive models that highlight the importance of cognitive and behavioral factors in both the development and maintenance of health anxiety, information seeking is such a behavioral factor (Salkovskis and Warwick, 1986; Salkovskis and Warwick, 2001; Taylor et al., 2004). A meta-analysis of 20 studies revealed that greater information-seeking behavior was associated with higher levels of health anxiety with moderate effect sizes (McMullan et al., 2019). Also, during periods of high risk, greater information-seeking is associated with higher levels of anxiety (Turner et al., 2006). A recent longitudinal study found that information-seeking predicted health anxiety at one-month follow-up during COVID-19 (Jagtap et al., 2021).

Stigma may be a cognitive factor of general health anxiety among COVID-19 patients and is prevalent in various types of infectious disease patients (Chi et al., 2014; Gardner and Moallef, 2015; Yuan et al., 2021). Individuals who have contracted COVID-19 may receive negative attitudes from society and experience difficulties in social interactions, including employment-related obstacles and isolation from family and friends (Al Eid et al., 2021). This may result in their internalization of these negative attitudes from society, self-devaluation and withdrawal from social activities (Yang et al., 2017). According to the minority-stress model, difficult social situations cause stress for minority individuals (e.g., perceived stigma, self-stigma), which accrues over time, resulting in long-term health deficits (Meyer, 2003). Several studies on patients with infectious diseases have demonstrated that perceived stigma could cause depression, anxiety, suicide, low self-esteem, resilience and help-seeking, and poor recovery and physical health (Chi et al., 2014; Alkathiri et al., 2022). The positive association between perceived stigma and health anxiety was also reported among people with chronic medical conditions (Dattilo et al., 2022). However, no studies have tested this association in people with COVID-19. Also, it is intriguing to explore whether COVID-19 symptoms could still lead to minority stress and stigma experience since the majority of people were infected in the recent wave of COVID-19.

In May 2023, the WHO emphasized that COVID-19 is an established and ongoing health issue (WHO. Statement on the fifteenth meeting of the IHR, 2005). In the last 28 days (17 April to 14 May 2023), nearly 2.6 million new cases and over 17,000 deaths were reported globally (WHO, 2023b). The latest data from the Chinese Centre for Disease Control and Prevention (CDC), released in April 2023, also highlighted that COVID-19 has not completely disappeared (Prevention CCfDCa, 2023). On the other hand, even though we have entered the post-pandemic phase, the long-term effects of COVID-19 and disease control policy (e.g., long COVID, mental and behavioral problems) may last at individual and societal levels. Therefore, it is important to explore the potential behavioral, social and health consequences (e.g., internet health information-seeking, perceived stigma, health anxiety) and the underlying mechanisms among people infected with COVID-19. The current study aims to: (1) describe the characteristics of COVID-19 symptoms (i.e., fever and cough) among individuals infected after China eased anti-COVID measures, and (2) test the relationships of COVID-19 symptoms with internet health information-seeking for COVID-19, perceived stigma, and health anxiety. It is hypothesized that (1) COVID-19 symptoms would be positively associated with online information seeking, perceived stigma, and health anxiety, and (2) internet health information-seeking for COVID-19 and perceived stigma would mediate the relationship between COVID-19 symptoms and health anxiety. The findings would provide valuable information for policymakers to evaluate and adjust the disease control policy and measures and for healthcare providers to understand the health concerns of people infected with COVID-19 and improve healthcare services.

## 2. Methods

### 2.1. Study design

**Figure 1:**
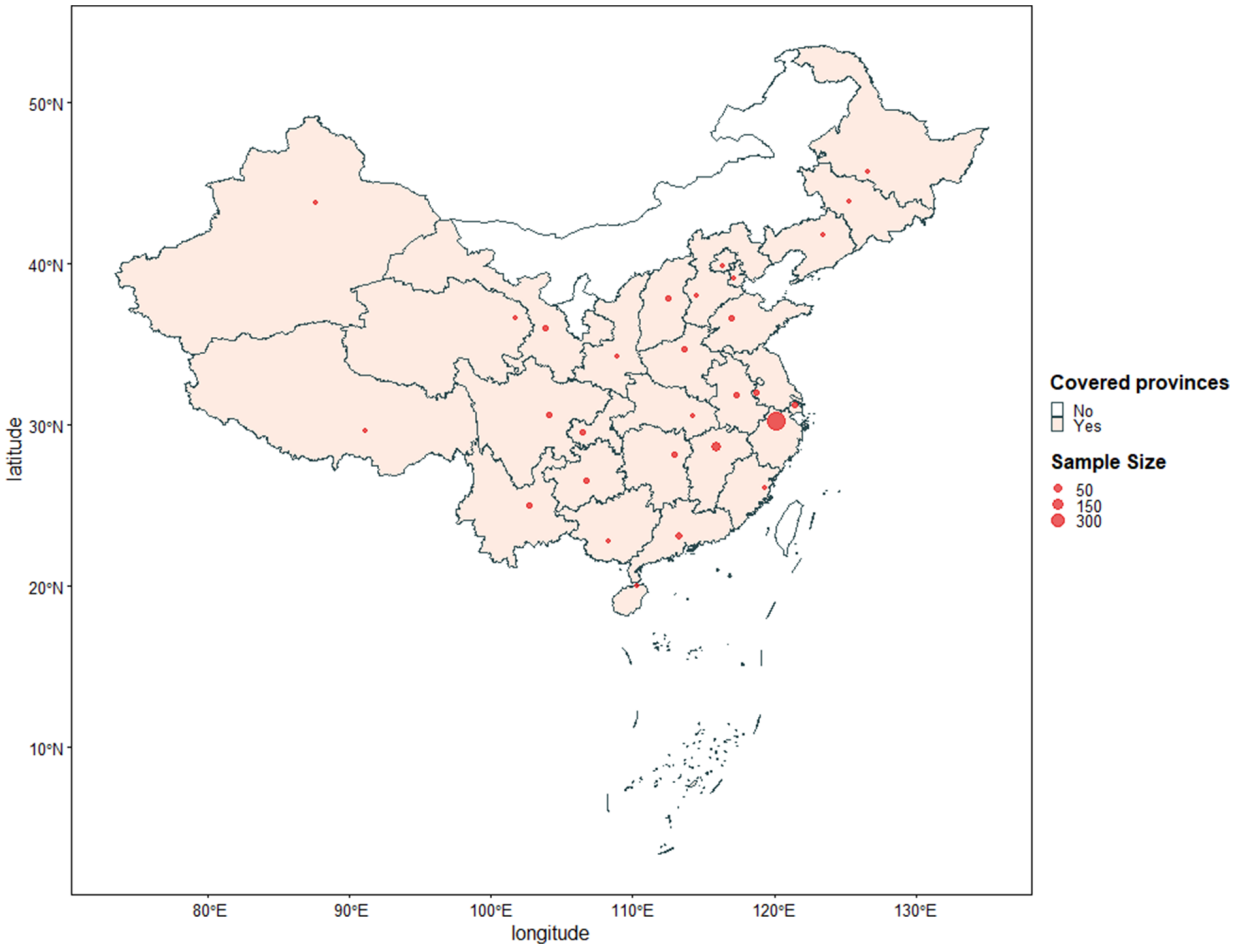
Data distribution in China.

This cross-sectional study was conducted within a month following the government’s relaxation of anti-COVID measures in December 2022, covering 25 provinces and four municipalities in China. Data distribution was shown in Figure 1. The data was collected through an anonymous online questionnaire among individuals who self-reported being infected with COVID-19 after the disease control relaxation. The online survey link was disseminated via WeChat. Participants were automatically offered a compensation of five RMB as a token of appreciation online after they submitted the completed survey. A total of 1,133 participants completed the questionnaire, with one invalid response excluded. Ethics approval was obtained from the corresponding author’s University Ethics Committee.

### 2.2. Measures

#### 2.2.1. COVID-19 symptoms

COVID-19 symptoms were assessed by participants’ self-report. Participants were asked whether they had experienced any of the listed symptoms since the government eased anti-COVID measures. Alternate options include fever, cough, shortness of breath, sore throat, fatigue, loss of taste and smell, nasal congestion, skeletal muscle pain, gastrointestinal reaction, and others. Those were mentioned in a systematic review of COVID-19 common symptoms (Alimohamadi et al., 2020). The total number of symptoms reported by each participant ranges from 0 to 10. Additionally, participants rated the severity of each symptom on a 5-point Likert scale, with a total score ranging from 0 to 50. A higher score indicates more severe symptoms. The reliability of the COVID-19 symptoms scale was found to be good in this study, with a Cronbach’s *α* of 0.86.

#### 2.2.2. Internet health information-seeking behavior

The four-item Internet health information-seeking behavior (IHISB) scale was used to evaluate Internet usage for health information-seeking for COVID-19. This measure is rated on a 7-point Likert scale, with responses ranging from “1 = strongly disagree” to “7 = strongly agree.” A higher total score indicates greater information-seeking online. IHISB has been previously validated among the Chinese population, yielding a Cronbach’s *α* of 0.75 (Liu et al., 2021). The Cronbach’s *α* was 0.94 in this sample.

#### 2.2.3. Perceived COVID-19 stigma

The COVID-19 Stigma Scale (COVID-SS) was used to assess the perceived stigma toward COVID-19. It consists of 11 items rated on a 5-point Likert scale (1 = never to 2 = always). Total scores range from 11 to 55, with higher scores indicating greater perceived stigmatization. COVID-SS has demonstrated good validity and reliability in previous research conducted among healthcare workers, with a Cronbach’s *α* coefficient of 0.91 (Al Houri et al., 2022). In the current study, COVID-SS showed good reliability (Cronbach’s *α* = 0.93).

#### 2.2.4. Health anxiety

Participants were asked to indicate to what extent they experienced: (1) worry about their health, (2) fear of becoming sick, and (3) perceived concerns about their health from their family and friends. Responses are rated on a 7-point Likert scale ranging “1 = strongly disagree” to “7 = strongly agree,” where high scores indicate greater severity of health anxiety. The health anxiety scale demonstrated good reliability with a Cronbach’s *α* coefficient of 0.82 among the Chinese population (Liu et al., 2021). In the current study, the health anxiety scale also showed satisfactory reliability (Cronbach’s *α* = 0.85).

#### 2.2.5. Background factors

Demographic information, including age, gender, education, type of work, health insurance, residence, and current infection status was self-reported by the participants and controlled as confounders.

### 2.3. Statistical analysis

Demographic characteristics were presented as mean [standard deviation (SD)] or frequency (%). Pearson correlation analysis and partial correlation analysis with adjusting for demographic factors were conducted. Confirmatory factor analysis (CFA) was used to test the measurement model. Structural equation modeling (SEM) was conducted to examine the proposed mediation model. Maximum Likelihood (ML) estimation was employed. The bias-corrected bootstrap with 5,000 times replications was used to estimate the 95% confidence interval (CI) of the path coefficients and indirect effects. The standardized direct effects of 0.1, 0.3, and 0.5 were considered small, medium, and large, respectively (Lt and Bentler, 1999). Indirect effects of 0.01, 0.09, and 0.25 were considered small, medium, and large, respectively (Mahmoudi et al., 2021). Models with *χ*^2^/df < 3, the Comparative Fit Index (CFI) > 0.90, the Tucker-Lewis Index (TLI) > 0.90, the Root Means Square Error of Approximation (RMSEA) < 0.08, and the Standardized Root Mean Square Residual (SRMR) < 0.08 were considered acceptable (Lt and Bentler, 1999; Mahmoudi et al., 2021). Statistical significance was considered when the two-tailed value of p was less than 0.05. R program (version 4.1.3) and Mplus (version 7.4) were used to perform all statistical analyses. Missing value was minimal and we did not further deal with it.

## 3. Results

### 3.1. Descriptive characteristics

The study included 1,132 participants (women, 67.6%), with a mean (SD) age of 28.12 (10.07) years. Over half of the participants (55.9%) had contracted COVID-19, while the others had recovered. Participants reported an average of 7 COVID-19 symptoms. Cough was the most common symptom (91.3%), followed by nasal congestion (89.1%) and fatigue (87.8%). The mean scores for IHISD, COVID-SS, and health anxiety were 19.16 (5.49), 19.87 (8.42), and 13.33 (4.18), respectively (Table 1).

**Table 1:** Demographic characteristics (*N* = 1,132).

Variables	*N* (%)
Age, mean (SD)	28.12 (10.07)
Gender	
Male	367 (32.4)
Female	765 (67.6)
Education	
Middle school or below	64 (5.7)
University	893 (78.9)
Postgraduate	175 (15.5)
Type of work	
Work that requires a high frequency of face-to-face contact with people	445 (39.3)
Work requiring a medium frequency of face-to-face contact with people	462 (40.8)
Work requiring a low frequency of face-to-face contact with people	38 (3.4)
No job	187 (16.5)
Health insurance	
No	53 (4.7)
Yes	1,079 (95.3)
Residence	
Urban	839 (74.1)
Rural area	293 (25.9)
Current infection status	
Infection symptoms have disappeared and the body has recovered	499 (44.1)
Been infected now	633 (55.9)
IHISB scores, mean (SD)	19.16 (5.49)
COVID-SS scores, mean (SD)	19.87 (8.42)
Health anxiety, mean (SD)	13.33 (4.18)
Severity of COVID-19 symptoms, mean (SD)	21.69 (10.50)
Number of COVID-19 symptoms, mean (SD)	7.33 (2.46)
Typical symptoms of infection	
Cough	1,034 (91.3)
Nasal congestion	1,009 (89.1)
Fatigue	994 (87.8)
Sore throat	972 (85.9)
Fever	952 (84.1)
Skeletal muscle pain	887 (78.4)
Gastrointestinal reaction	743 (65.6)
Shortness of breath	668 (59.0)
Loss of taste and smell	611 (54.0)
Others	425 (37.5)

IHISB, internet health information seeking behavior; COVID-SS, COVID-19 stigma scale; SD, standard deviation.

### 3.2. Correlation analyses

As Table 2 showed, all the studied variables were positively associated with each other with and without adjusting for covariates (all *p* < 0.01).

**Table 2:** Correlations between the studied variables.

	1	2	3	4	5
1. Number of COVID-19 symptoms	–	0.82***	0.12***	0.24***	0.15***
2. Severity of COVID-19 symptoms	0.81***	–	0.18***	0.26***	0.20***
3. IHISB scores	0.10**	0.15***	–	0.17***	0.77***
4. COVID-SS scores	0.25***	0.26***	0.17***	–	0.27***
5. Health anxiety	0.13***	0.18***	0.77***	0.27***	–

The lower triangle was partial correlation analyses with adjusting for age, gender, education, type of work, residence, health insurance, current infection status.

IHISB, internet health information seeking behavior; COVID-SS, COVID-19 stigma scale.

***p < 0.001; **p < 0.01; *p < 0.05.

### 3.3. Structural equation modeling

The measurement model showed a good fit with, *χ*^2^/df < 1, CFI = 0.99, TLI = 1.00, RMSEA <0.01, SRMR <0.01. The number and severity of COVID-19 symptoms showed significant loadings on the latent variable of COVID-19 symptoms, with standardized factor loadings of 0.85 and 0.97, respectively (both *p* < 0.001).

**Figure 2:**
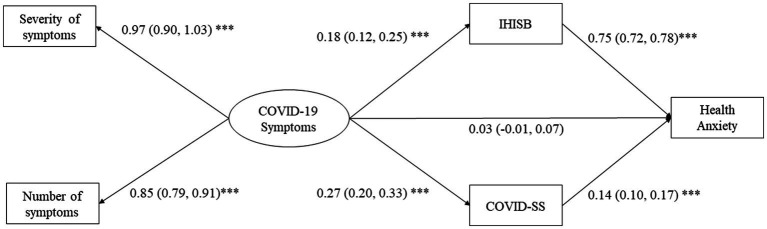
Mediation roles of IHISB and COVID-SS between COVID-19 symptoms and health anxiety. Results were shown as coefficients (95% CI). All paths to Health Anxiety were adjusted age, gender, education, type of work, residence, health insurance, current infection status. ***p<0.001; **p<0.01. IHISB: internet health information seeking behaviour; COVID-SS: COVID-19 stigma scale; CI: confidence interval.

The structural model fit well: *χ*^2^/df = 2.83, CFI = 0.95, TLI = 0.93, RMSEA = 0.05, SRMR = 0.04. As shown in Figure 2 and Table 3, the direct effect of COVID-19 symptoms on health anxiety was not significant. The indirect effects of IHISB (*β* = 0.14, 95%CI = 0.09–0.20) and COVID-SS (*β* = 0.04, 95%CI = 0.02–0.06) were significant, respectively. Thus, their full mediation effects were demonstrated.

**Table 3:** Mediation effects of IHISB and COVID-SS between COVID-19 symptoms and health anxiety.

	Effect	*p*
Total effect of COVID-19 symptoms on health anxiety	0.21 (0.14, 0.27)	<0.001
Specific indirect effect		
COVID-19 symptoms -- > IHISB -- > health anxiety	0.14 (0.09, 0.19)	<0.001
COVID-19 symptoms -- > COVID-SS -- > health anxiety	0.04 (0.03, 0.05)	<0.001
Direct effect		
COVID-19 symptoms -- > health anxiety	0.03 (−0.01, 0.07)	0.166
Model fit information		
*χ*^2^/df	2.83	
RMSEA (Root mean square error of approximation)	0.05	–
SRMR (Standardized root mean square residual)	0.04	–
CFI	0.95	–
TLI	0.93	–

All paths to health anxiety were adjusted for age, gender, education, type of work, residence, health insurance, current infection status.

IHISB, internet health information seeking behavior; COVID-SS, COVID-19 stigma scale; df, degrees of freedom; RMSEA, root mean square error of approximation; SRMR, standardized root mean square residual; CFI, comparative fit index; TLI, tucker lewis index.

## 4. Discussion

Our sample reported an average of seven symptoms, with cough, nasal congestion, and fatigue being the most commonly reported by people infected in the recent wave of COVID-19. It is slightly different from those reported at the beginning of the pandemic in 2020, where fever, cough, and fatigue were the most common symptoms (Alimohamadi et al., 2020). Possible reasons for this difference include the emerging new variant strains and the fact that most residents in China had been vaccinated. Indeed, people who suffered from a greater number or severity of symptoms might experience more daily life disruptions and pains, and tend to consider their immune function and physical quality poor, which can lead to more anxiety emotions and long-term concerns about general health.

As we hypothesized, COVID-19 symptoms were positively associated with general health anxiety. We further identified that behavioral (IHISB) and cognitive mechanisms (COVID-SS) could fully explain this association in our mediation model. IHISB appeared to be an important mediator as it showed a medium to large indirect effect size. People who suffered from a greater number or severity of symptoms might experience more daily life disruptions and pains, and thus they are more motivated to seek more information about COVID-19. In this digital age, IHISB plays an important role in enhancing health literacy in the general public and facilitating disease management and self-aid, especially when local medical services are limited (Jamal et al., 2015; Liu, 2020). However, our results highlight its potential dark side that might induce health anxiety among people suffering from COVID-19 symptoms. A plausible reason may be that the beneficial or harmful effects of IHISB depend on information quality, information sources, and individuals’ online-health literacy. Previous studies have suggested that not all online information is true or accurate which can lead to catastrophic beliefs and panic in the general public (Jagtap et al., 2021). Especially during COVID-19, the general public is exposed to extensive negative news (e.g., severe cases, deaths, limited medical services) and inaccurate information. The WHO also suggested that an overwhelming amount of information (both true and false) during a pandemic can lead to anxiety and potentially harmful behaviors. To prevent the negative consequences of IHISB, it is important to rely on trusted online sources of information, such as the WHO, the CDC, and other reputable health organizations, and learn to critically evaluate the information.

COVID-SS had a small to medium indirect effect size. Participants with greater COVID-19 symptoms were more likely to perceive stigma. This finding is consistent with previous studies on COVID-19 (Lin et al., 2021) and other infectious diseases (Chi et al., 2014; Gardner and Moallef, 2015). Infectious diseases can be associated with fear and discrimination in the general public (e.g., blame, social exclusion) and even self-stigmatization among people with the disease (e.g., shame for their perceived carelessness or exposure to the virus, self-blame for spreading the virus, especially to their family and friends). In turn, we found that such a stigma experience could increase one’s anxiety about general health, highlighting the importance of minority stress in mental health and perceived health status. Similarly, studies in patients with chronic diseases, such as inflammatory bowel disease, asthma, and diabetes, have demonstrated that those who perceived greater illness stigma experienced greater health anxiety (Taft and Keefer, 2016; Dattilo et al., 2022). These findings extend the application of the minority stress theory to COVID-19 which is an emerging health threat that the majority have been infected with. It would be informative to have in-depth interviews among people infected with COVID-19 to understand whether or why they feel powerless and how they perceive the risk of reinfection, long COVID-19 symptoms, and their health and react to these concerns. It is also important to monitor the long-term effects of COVID-19 symptoms and stigma on health concerns and behavioral responses (e.g., help-seeking).

Our findings have important practical implications to minimize the long-term health and social effects and for better policy planning for future potential pandemics. Health authorities and health professionals should better manage official channels and health-related information on media to facilitate public health communication and reduce stigma. Emotional support and mental health service to patients with severe symptoms should be provided. More intensive psychotherapies, such as cognitive behavioral therapy, may be particularly helpful for those who experienced significant stigma and health anxiety.

### 4.1. Limitations

This study has several limitations that should be considered. First, this is a cross-sectional study, which limits the ability to infer causality. Future longitudinal studies are needed to determine whether COVID-19 symptoms result in changes in IHISB and perceived stigma, which can predict changes in health anxiety over time. Second, the study was conducted among young adults, which limits its generalizability to other age groups. Further research is recommended to investigate whether these findings are replicated in older adults. Last but not least, household income, as one of the critical confounders, was not properly adjusted due to the lack of information. Education, medical insurance, and living area were adjusted to reduce potential bias. Future studies should consider including household income to better understand the unbiased effects.

## 5. Conclusion

To our knowledge, this is the first study to test whether and how the number and severity of COVID-19 symptoms would affect health anxiety. We found that COVID-19 symptoms were positively associated with health anxiety via the mediation effects of internet health information-seeking behavior and COVID-19 stigma. Interventions for health anxiety reduction during and after pandemics should target improving the quality of online health information, enhancing individuals’ online healthy literacy, and reducing stigma.

## Data availability statement

The raw data supporting the conclusions of this article will be made available by the authors, without undue reservation.

## Ethics statement

The studies involving human participants were reviewed and approved by the Wenzhou Medical University. Written informed consent to participate in this study was provided by the participants’ legal guardian/next of kin.

## Author contributions

QL was responsible for analyzing the data, interpreting the results, and writing the draft. XY, GZ, and XW conceptualized the study framework. HZ assisted with literature review. GZ was responsible for the study design and data collection. XY provided critical feedback on data analyses, data interpretation, and manuscript writing. ND, WZ, WT, JH, MD, and HH helped with data collection. All authors contributed to the article and approved the submitted version.

## Conflict of interest

The authors declare that the research was conducted in the absence of any commercial or financial relationships that could be construed as a potential conflict of interest.

## Publisher’s note

All claims expressed in this article are solely those of the authors and do not necessarily represent those of their affiliated organizations, or those of the publisher, the editors and the reviewers. Any product that may be evaluated in this article, or claim that may be made by its manufacturer, is not guaranteed or endorsed by the publisher.
